# ‘Relax and Repair’ to restrain aging

**DOI:** 10.18632/aging.100399

**Published:** 2011-10-30

**Authors:** Vaidehi Krishnan, Baohua Liu, Zhongjun Zhou

**Affiliations:** ^1^ Department of Biochemistry, LKS Faculty of Medicine, The University of Hong Kong, Hong Kong; ^2^ Cancer Science Institute of Singapore, National University of Singapore, Singapore; ^3^ Shenzhen Institute of Research and Innovation, The University of Hong Kong, Shenzhen, China

**Keywords:** lamin A, DNA repair, chromatin, prelamin A, premature aging, histone acetylation, epigenetics, senescence, Zmpste24, HGPS

## Abstract

The maintenance of genomic integrity requires the precise identification and repair of DNA damage. Since DNA is packaged and condensed into higher order chromatin, the events associated with DNA damage recognition and repair are orchestrated within the layers of chromatin. Very similar to transcription, during DNA repair, chromatin remodelling events and histone modifications act in concert to ‘open’ and relax chromatin structure so that repair proteins can gain access to DNA damage sites. One such histone mark critical for maintaining chromatin structure is acetylated lysine 16 of histone H4 (AcH4K16), a modification that can disrupt higher order chromatin organization and convert it into a more ‘relaxed’ configuration. We have recently shown that impaired H4K16 acetylation delays the accumulation of repair proteins to double strand break (DSB) sites which results in defective genome maintenance and accelerated aging in a laminopathy-based premature aging mouse model. These results support the idea that epigenetic factors may directly contribute to genomic instability and aging by regulating the efficiency of DSB repair. In this article, the interplay between epigenetic misregulation, defective DNA repair and aging is discussed.

## Organization of chromatin

The genomes of organisms are organized in the form of a fundamental structure called chromatin in which the repeating nucleosomes form the basic unit. The nucleosome consists of 147 bp of DNA wound 1.7 times around an octamer composed of the four core histones, H2A, H2B, H3 and H4. Multiple nucleosomes are further linked by DNA stretches that are occupied by linker histone H1, to form the 10-nm fibre or ‘beads on a string’ type of arrangement. Chromatin fibres undergo compaction through intramolecular nucleosome-nucleosome interactions to form the 30 nM chromatin fibres. At the next level of organization, chromatin is further stacked and folded to give rise to 100-400 nm interphase chromatin fibres. The DNA that is eventually folded into chromosomes has already undergone compaction by about 10,000 fold. The packaging of DNA into condensed and often inaccessible chromatin imposes a significant constraint for the efficient repair of DNA double strand breaks (DSBs). Recent efforts have been directed towards understanding how the DNA repair proteins gain access to and repair, when DNA damage is embedded within chromatin fibres.

## Chromatin structure and DNA repair

Cells utilize two distinct mechanisms to modulate chromatin dynamics during DNA repair. These mechanisms include the post translational modifications of histones and ATP-dependent chromatin remodelling. According to the ‘histone code hypothesis’, the biological outcome of histone modifications is manifested by providing a signalling platform for the recruitment of downstream effector and reader protein or by the physical modulation of chromatin structure [1]. Accordingly, histones within chromosomes are subjected to several forms of post translational modifications such as phosphorylation, ubiquitination, methylation, and acetylation and these modifications can either create or eliminate binding sites for non-histone proteins that mediate DNA repair or modify chromatin structure [2]. The earliest identified and one of the most important histone modifications during DNA repair is the phosphorylation of histone H2AX at the C-terminal residue corresponding to Ser139 (γ-H2AX) by the key DNA damage-responsive kinase, ATM [3].During DSB repair, phosphorylated H2AX forms a specialised chromatin compartment capable of recruiting and retaining DNA repair factors [4]. H2AX phosphorylation spreads over a 2 Mb domain on each side of the DSB, and acts as a docking site for several DNA repair proteins such as the mediator, MDC1. Thus, H2AX phosphorylation acts as an important cue for the stable retention of DNA repair proteins which form microscopically discernible foci, called as irradiation-induced foci (IRIF) [5]. It is now recognized that apart from phosphorylation, H2AX is monoubiquitinated and later di and poly-ubiquitinated in a DNA damage-dependent manner. According to current understanding, several ubiquitin ligases including RNF8, RNF168, RNF2, Bmi1 and Herc2 are responsible for the completion ubiquitination of γ-H2AX [6]. In turn, ubiquitinated histones promote the recruitment of DNA repair proteins, Brca1and 53BP1 which directly participate in the repair of DSBs by homologous recombination (HR) or non-homologous end joining mechanisms (NHEJ). Apart from histone ubiquitination, histone methylation also plays an important role in DSB repair processes. Trimethylated H3K9 is known to be an important component of heterochromatin and the heterochromatin 1 proteins, HP1 α,β,δ, bind to trimethylated H3K9, contributing to heterochromatin maintenance. Upon DSB induction, HP1 β is phosphorylated in a casein kinase-dependent manner, which promotes HP1 β dissociation and γ -H2AX phosphorylation [7]. Following HP1 β dissociation, the MYST family histone acetyltransferase (HAT) Tip60, is recruited to trimethylated H3K9 resulting in the stimulation of its HAT activity [8]. In other examples, constitutive dimethylation of histone H3K79 by Dot1L and DNA damage-inducible H4K20 dimethylation by MMSET also provide a recruitment platform for the DSB repair protein, 53BP1 [9, 10]. Thus, in general, histone methylation mainly provides binding sites for the direct recruitment for downstream repair proteins.

It is well known that histone acetylation is associated with a more ‘open’ configuration of DNA, because histone acetylation imparts a negative charge that causes the charge repulsion of negatively charged DNA. The acetylation of H2AX at K5 position by Tip60 regulates the ubiquitination of H2AX at K119 and enhances chromatin dynamics [11]. Tip60-TRRAP mediated histone H4 acetylation is also required for efficient recruitment of repair proteins and HR [12]. Since the requirement of Tip60-TRRAP could be overridden by forced chromatin relaxation using histone deacetylases (HDAC) inhibitors, it was proposed that the main role of histone acetylation was to improve chromatin accessibility. In a similar mechanism, Mof, another MYST family HAT, mediates H4K16 acetylation and controls higher order chromatin configuration to promote repair protein recruitment (as discussed below). Another example is the acetylation of H3K14 which increases after irradiation in a HMGN1-dependent manner. In this study, H3K14 acetylation was shown to regulate ATM activation and since the requirement for HGMN1 could be overcome by inducing chromatin relaxation using HDAC inhibitors, it was suggested that HGMN1 regulates higher order chromatin structure during DNA repair [13]. The recurring theme in these experiments that forced chromatin relaxation could bypass the requirement for histone acetylation, suggests that promoting chromatin accessibility was likely to be one of the principle role of histone acetylation in DSB repair.

The direct recruitment of ATP-dependent chromatin remodelling complexes that enzymatically modulate chromatin structure is another important mechanism in DNA repair. Chromatin remodelling complexes use ATP hydrolysis to increase accessibility of nucleosomal DNA by repositioning nucleosomes or by altering nucleosomal composition [14]. There are four main families of mammalian chromatin remodelling complexes: the SWI/SNF (switching defective/sucrose non-fermenting) family, the NuRD (nucleosome remodelling and deacetylation)/Mi-2/CHD (chromo-domain helicase DNA binding family, INO80 (inositol requiring 80) family and the ISWI (imitation-SWI) family of complexes. An idea that has emerged in recent years is that DNA damage-modified histones provide targeting sites for the recruitment of chromatin remodelling complexes. Thus, specificity is achieved by chromatin-interacting domains that bind to modified histones. In pioneering studies conducted in budding yeast, γ-H2AX was shown to provide binding sites for the recruitment of ATP-dependent INO80, and histone acetylating NuA4 at the site of a DSB [15, 16]. Likewise, both members of SWI/SNF family of ATPases, BRM and BRG1 directly interact with acetylated H3 residues in γ-H2AX containing nucleosomes [17]. This interaction increases the recruitment of the HAT, GCN5, which promotes further efficient induction of γ-H2AX after irradiation. SWI/SNF is targeted to DSBs by interaction with an early DNA damage responsive protein BRIT1/MCPH1, which then promote the recruitment of repair factors such as MDC1, Rad51, Ku70, RPA and NBS1 to DSBs [18]. The mammalian ISWI remodelling complexes containing the SNF2H or SNF2L ATPases mediate nucleosome sliding and histone replacement and are recruited to DSBs in micro-irradiation experiments [19]. SNF2H promotes DNA repair by HR and its depletion impairs RPA, Brca1 and Rad51 foci formation [20]. Mammalian INO80 chromatin remodelling complexes contain the INO80 ATPase and several other subunits which form a complex with the polycomb transcription factor YY1. INO80 is recruited to laser-generated DSBs and the loss of YYI or INO80 leads to chromosome aberrations and defective repair by HR [21]. Mammalian CHD remodelling complexes slide or eject histones and several members of this family such as the CHD1-like protein ALC1 and CHD2 have been proposed to play a role in the DSB repair by NHEJ [22, 23]. Recent studies have implicated the CHD4 ATPase in multiple stages of the DDR. CHD4 is recruited to DSB lesions and its depletion led to reduction in DNA damage-induced histone ubiquitination and defective recruitment of Brca1 and RNF168 [24, 25]. The loss of CDH4 or other NuRD components also led to structural defects in the chromatin and increased accumulation of spontaneous DNA damage [26].

In other examples involving chromatin structure and DNA repair, DSB repair in the heterochromatin requires specialized mechanisms to promote access to repair proteins because heterochromatin is structurally inhibitory to DNA repair owing to its condensed structure. ATM-dependent phosphorylation of the co-repressor Kap1 was shown to allow localized and transient chromatin relaxation at regions of heterochromatin and promote the recruitment of DNA repair proteins [27]. Likewise, it has been shown that the PWWP domain-containing protein EXPAND1 accumulates in IRIF in a H2AX, MDC1, RNF8 and 53BP1-dependent manner and increases chromatin accessibility after DNA damage [28]. Thus, DSB recognition and repair are intimately associated with chromatin remodelling and histone modification events.

## Laminopathy-based premature aging

Amongst the most prominent phenotypes associated with defective DSB repair in both humans and mice, is the onset of accelerated aging (progeria). In several correlative studies, γ -H2AX foci containing senescent cells increase with age in humans, mice and primates, leading to the model that inefficient DSB processing and repair can activate cellular senescence pathways and initiate premature aging [29-31]. This notion is strengthened with the observation that knockout-mice defective for DSB repair often show an accelerated aging phenotype and conversely, defective DNA repair is a common phenotype in human patients suffering from premature aging (progeria) syndromes [32-33].

**Figure 1:**
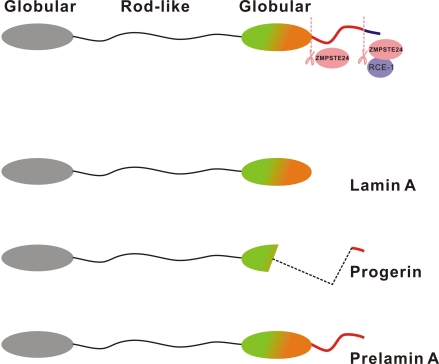
Structure of prelamin A, lamin A and progerin Lamin A is synthesized as a 74-kDa precursor, prelamin A. The C-terminal CaaX motif of prelamin A undergoes a series of posttranslational modifications including CaaX processing (farnesylation, aaX cleavage and carboxylmethylation), followed by endoproteolytic cleavage by Zmpste24. Zmpste24 is responsible for the sequential proteolytic cleavage and processing of prelamin A into mature lamin A (70-kDa). The point mutation identified from HGPS patients, results in the activation of an aberrant cryptic splice site causing the deletion of a 50 amino acid region from the C-terminal end of prelamin A. Hence, prelamin A cannot undergo complete processing into mature lamin A in HGPS patients, and a truncated protein called as progerin accumulates in cells.

In our earlier study involving human Hutchinson Gilford progeria syndrome (HGPS) patient fibroblasts, defective DNA repair and genomic instability was demonstrated [34]. Clinical manifestations in HGPS patients include accelerated aging symptoms like hair loss and greying, atherosclerosis, short stature, sculpted nose, reduced subcutaneous fat, decreased bone density and cardiovascular diseases. HGPS patients die at an average age of 13 years due to complications related to atherosclerosis. A single *de novo* point mutation of nuclear protein lamin A gene at position 1824 (C to T) in exon 11, was found to be predominantly responsible for this syndrome. Lamin A is synthesized as the precursor protein, prelamin A which is modified at its carboxyl-terminal through a series of post translational modifications. The modifications include farnesylation of the cysteine in the C-terminus CAAX (C-cysteine, A-aliphatic, X-other amino acid)-motif, followed by proteolytic cleavage of the AAX-peptide, and methylation of the farnesylated cysteine. The metalloproteinase, Zmpste24, is responsible for the sequential proteolytic cleavage of prelamin A into functional and mature lamin A. The point mutation identified from HGPS patients results in the activation of an aberrant cryptic splice site causing the deletion of a 50 amino acid region from the C-terminal end of prelamin A [35]. Since the deletion harbours a cleavage site for the enzyme Zmpste24, prelamin A cannot undergo complete processing into mature lamin A in HGPS patients, and a truncated protein that lacks amino acids 607-656 (called as progerin) accumulates in cells. Thus, the unprocessed forms of lamin A, progerin and prelamin A accumulate in HGPS and Zmpste24-null cells respectively (Figure 1).

Remarkably, human HGPS premature aging phenotypes can be recapitulated in Zmpste24-null mice indicating that the presence of 18 extra amino acid tail of prelamin A is responsible for premature aging [36]. At the cellular level, accumulation of prelamin A/progerin leads to phenotypes such as nuclear shape abnormalities, nuclear blebbing, loss of hetero-chromatin, epigenetic alterations and early cellular senescence [37-39]. Interestingly, heterozygosity for lamin A (*Zmpste24−/− lmna+/−*) largely ameliorated the progeria-like phenotypes in Zmpste24-deficient mice, suggesting that reducing prelamin A concentration by 50% was enough to reduce accelerated aging phenotypes [40].

The observation that Zmpste24-null and human HGPS patient cells have genomic instability was important in understanding the mechanisms contributing to premature aging [34]. Fibroblasts isolated from HGPS patients and Zmpste24-null MEFs accumulated γ-H2AX foci in culture and were sensitive to a variety of DNA damaging agents. Furthermore, karyotyping analysis revealed aneuploidy in the bone marrow of Zmpste24-null mice. HGPS and Zmpste24-null cells were also found defective for DNA repair, in assays using DSB repair reporter cassettes. Based on these observations, HGPS was classified together with other human progeroid syndromes as a genomic instability-associated disorder. At a more molecular level, the recruitment of DNA repair proteins 53BP1 and Rad51 was significantly delayed after DSB induction in Zmpste24-null and HGPS fibroblasts. In subsequent studies, other repair proteins such as MDC1 and Mre11 also showed impaired recruitment, suggesting that the early response to DNA damage was dramatically inhibited in the presence of mutant lamin A [41-46]. Together, these data have led to the idea that prelamin A/progerin accumulation interferes with the loading of DNA repair proteins to DSB sites and that, as a consequence, irreparable DNA damage remains which fuels a cycle of chronic DNA damage response, premature senescence and aging.

## Down regulation of Mof-dependent H4K16 acetylation in Zmpste24-null fibroblasts

Lamin A is a structural constituent of a subnuclear compartment called as the nuclear matrix [47]. The nuclear matrix is a nuclear subcellular compartment that is thought to provide a scaffold to facilitate chromatin organization and transcriptional regulation [48]. The nuclear matrix is recognized as a distinct subcellular compartment constituted of detergent and DNase-insoluble proteins.The observation that lamin A mutants are defective for DNA repair was surprising given that there was no precedent of a nuclear matrix-associated structural protein capable of regulating DNA repair.

In a recent study, we have investigated the mechanism underlying defective DNA repair and premature aging in Zmpste24-null cells and identified a role for histone H4K16 hypoacetylation in aging [49]. We hypothesized that prelamin A accumulation might disrupt DNA repair if epigenetic histone modifications associated with DNA repair are misregulated. To test this hypothesis, early passage mouse embryonic fibroblasts (MEFs) isolated from Zmpste24 wild-type and null mice were systematically screened with specific antibodies directed against histone modifications. In order to ensure that identified epigenetic differences arose as a primary consequence of prelamin A expression, experiments were performed using early passage MEFs or prelamin A-transfected cell lines. Based on this analysis, prelamin A-expressing cells were found defective in the acetylation of H4 at lysine 16 residue (H4K16). Since the defect in H4K16 acetylation temporally preceded the onset of cellular senescence, it was rationalized that H4K16 hypoacetylation might be responsible for the early cellular senescence phenotype of Zmpste24-null cells.

Using a combination of imaging techniques and biochemical fractionation, it was found that Mof, the principle histone H4K16 acetyltransferase localizes to the nuclear matrix in wild-type cells. However, in prelamin A-expressing cells, the nuclear matrix association of Mof was found severely reduced. At the molecular level, lamin A co-immunoprecipitates with Mof, whereas prelamin A had several folds lower binding with Mof. These observations implied that the 18 amino acid extra C-terminal tail of unprocessable prelamin A that is lacking in mature lamin A, prevented Mof binding and resultantly, the association of Mof with the nuclear matrix was reduced causing histone acetylation defects.

## H4K16 acetylation and DSB repair

How do reduced global H4K16 acetylation levels affect chromatin structure? In elegant work by Shogren-Knaak et al., the incorporation of acetylated K16 of H4 into nucleosomal arrays was demonstrated to impede the ability of chromatin to form cross-fibre interactions and this converted chromatin into an ‘open’ conformation [50]. In crystallization studies, it was observed that N- terminal of H4 tail of one mononucleosome interacted with the H2A/H2B of an adjacent mononucleosome, suggesting that this interaction might mediate chromatin compaction. Using chemical ligation, histone H4 that uniformly acetylated only at lysine 16 was prepared and intramolecular chromatin compaction was studied. Strikingly, these studies revealed that upon H4K16 acetylation, chromatin fibres could not achieve the fully compacted 30 nm fibre state adopted by unacetylated arrays.

**Figure 2:**
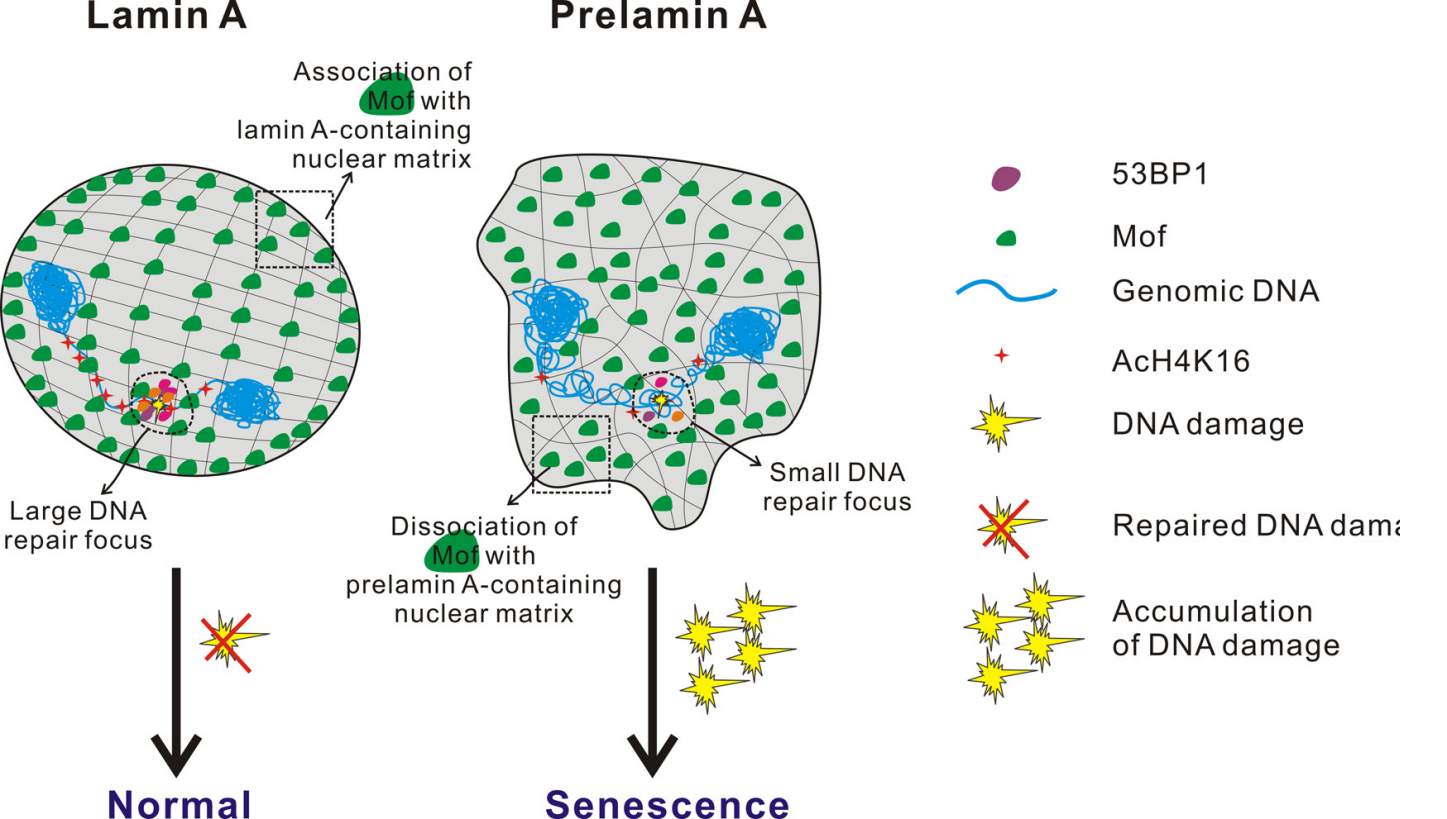
Model showing the relationship between impaired histone acetylation, defective DSB repair and pre-mature aging H4K16 acetylation impedes the ability of chromatin to form cross-fibre interactions and this converts chromatin into a ‘relaxed’ conformation. Mof, a MYST family histone acetyltransferase, is the enzyme mainly involved in acetylation of H4 at K16 position in mammalian cells. The 18 amino acid C-terminal tail of prelamin A prevents the proper association of Mof to the nuclear matrix leading to Mof mislocalization and the hypoacetylation of histone H4K16. Defective H4K16 acetylation, in turn, results in global chromatin compaction and the inability to assume the chromatin conformation required for repair process access. Consequently, the delayed recruitment of repair proteins to sites of DSBs causes the accumulation of irreparable DNA damage, chronic DNA damage response, early cellular senescence and premature aging.

As an extension of this idea, it was rationalised that global reduction of H4K16 acetylation may result in genome-wide chromatin compaction in Zmpste24-null cells, which has to be relieved first before repair proteins such as 53BP1 gain access to DNA damage sites. This model was tested by the re-introduction of Mof, which elevated H4K16 acetylation levels and dramatically promoted the recruitment of 53BP1 to sites of DSBs in Zmpste24-null cells. Consistently, as predicted, Mof knockdown had an opposite effect in that cellular senescence and accumulation of irreparable DNA damage exacerbated even further. Thus, an inverse correlation could be drawn between Mof function, DNA repair and premature senescence (Model summarized in Figure 2).

The relationship between Mof, chromatin structure and DNA repair has been further expounded in three recent independent studies. Owing to global compaction of the genome, the chromatin structure of Mof-null MEFs was altered in a way such that the cells became refractory to DNA damage signalling and repair protein recruitment. In the study by Li *et al*, severe G(2)/M cell cycle arrest, massive chromosome aberrations, and defects in ionizing radiation-induced DNA repair were observed in Mof-null cells [51]. The recruitment of repair mediator protein Mdc1, 53BP1 and Brca1 to DNA damage foci was completely abolished in the absence of Mof [51]. Importantly, the interaction between Mdc1 and γ-H2AX was shown to require H4K16 acetylation and an acidic pocket on H2AX, suggesting that inter and intranucleosomal interaction between H4 and H2AX was required to establish the chromatin configuration conducive for MDC1 association with γ-H2AX. Indeed, H2AX acidic patch mutants could not interact with acetylated H4K16 and thus failed to establish the chromatin structure required for MDC1 recruitment. In another study, MOF depletion also greatly decreased DSB repair by both NHEJ and HR [52]. A purkinje cell-specific conditional Mof knock out mouse displayed impaired motor coordination, ataxia, a backward-walking phenotype, and a reduced life span and some of these phenotypes were very similar to the cerebellar dysfunction observed in Ataxia-telangiectasia (AT) patients [53]. Together, these studies highlight that Mof is an important mediator of the DNA damage response.

Interestingly, upon careful analysis it is apparent that H4K16 acetylation levels begin to increase only at about 60 minutes post-irradiation (our unpublished observations), while it is known that the recruitment of repair proteins such as 53BP1 and MDC1 occurs temporally at earlier time points after irradiation. Since the recruitment of DNA repair proteins is found defective in Mof-null cells even at early time points after irradiation, it indicates that both basal as well as the DNA damage inducible-H4K16 acetylation levels are important to create the chromatin structure conducive for DNA damage recognition, DSB repair protein recruitment and further signalling.

## HDAC inhibition and DNA repair

A simplified view of the above model is that promoting chromatin accessibility by increasing histone acetylation levels can improve DNA repair by increasing the recruitment of DNA repair proteins. HDAC inhibitors prove useful for this purpose, since they are well-established as anti-cancer agents and several pharmacological agents are validated to increase histone acetylation levels [54]. Based on the rationale that HDAC inhibitors can promote DNA damage recognition and repair by promoting global chromatin relaxation, it was tested whether cellular senescence and premature aging phenotypes may be attenuated in Zmpste24-null cells upon HDAC inhibition. Interestingly, when Zmpste24-null cells were treated with HDAC inhibitors, sodium butyrate or trichostatin A, a significant reduction in the accumulation of unrepaired DSBs and an overall improvement in cell survival after DNA damage were noticed. Importantly, when Zmpste24-null mice were fed with sodium butyrate, a modest extension of life span and amelioration of premature aging phenotypes was observed, suggesting that HDAC inhibition might have a therapeutic potential. It is certainly possible that HDAC inhibitors may be non-specific in their action and thus it might be difficult to establish whether the extension of longevity is due to restoration of histone acetylation levels or due to other indirect benefits. Secondly, the dosage of HDAC inhibitors have to be carefully titrated since an excessive concentration might lead to toxicity. Despite these caveats, there are now several studies reporting the beneficial effects of HDAC inhibitors on aging. Recently, HDAC inhibitors were shown to improve DNA repair in an oncogene-induced senescence model by causing chromatin relaxation [55]. In other studies, HDAC inhibitors have been shown to increase learning ability, delay age-dependent neurodegeneration, delay Alzheimer's disease progression in mouse models, accelerate age-associated osteogenesis, and increase life span of worms in a dietary restriction model [56-60]. It still needs to be established if the therapeutic benefits of HDAC inhibitors in these model systems are linked to their ability to directly promote DNA repair by the relaxation of chromatin structure.

## H4K16 acetylation and physiological aging

Zmpste24-null mice or human HGPS cells have been considered as segmental progeroid syndromes, in that they only partially recapitulate the phenotypes associated with normal aging. Hence, it was believed that experiments conducted on premature aging syndromes may not accurately reflect physiological aging. This view is gradually changing owing to efforts made to detect progerin or prelamin A accumulation during normal aging. In the first such report, rare fibroblasts cultured from elderly individuals were found to exhibit nuclear abnormalities similar to HGPS cells [61]. Progerin transcripts and progerin protein could be detected from cells obtained from healthy individuals and more importantly the specific knockdown of progerin rescued age-associated nuclear deformities. In another study, the technical difficulty associated with progerin detection was overcome through the development of a progerin-specific antibody. Using this antibody, the progerin-positive fibroblasts were seen to increase in elderly skin [62]. Both prelamin A and progerin were also observed to accumulate in human vascular aging [63]. In a more recent study, a link between telomere dysfunction and progerin accumulation was established during normal aging. It was suggested that telomere shortening increased the production of progerin in normal cells and progerin and telomere dysfunction collaborate to trigger cellular senescence during normal aging [64].

The above studies raise the exciting possibility that Zmpste24-null/HGPS fibroblasts may offer a simple model to decode the molecular defects contributing to normal aging. Interestingly, in a study by Sedelnikova *et al*, genomic instability associated with normal aging was found associated with delayed recruitment of repair proteins to sites of DSBs [65]. Since this observation similar to what is observed in Zmpste24-null and HGPS fibroblasts, it is possible that H4K16 hypoacetylation can alter chromatin structure and reduce the efficiency of DNA repair during physiological aging as well. Consistently, global histone H4K16 acetylation depleted in an age-dependent manner in many tissues isolated from ‘old’ wild type mice. In another independent study, H4K16 acetylation levels were found to be higher in cell lines where life span has been prolonged by ectopic expression of hTERT [66]. An age-dependent reduction in H4K16 acetylation levels was also demonstrated using human oocytes [67]. Put together, these observations raise the possibility that H4K16 hypoacetylation associated with normal aging can contribute to genomic instability, by reducing the efficiency of DSB repair. Certainly, further investigations are required along these lines to address this hypothesis.

In contrast to the above model, global H4K16 acetylation levels were found to increase in a Sir2-dependent manner during yeast replicative aging. In a study by Dang *et al*, ‘old’ yeast cells were found to have increased amounts of H4K16 acetylation, due to decreased abundance of Sir2 deacetylase [68]. The increased levels of H4K16 acetylation correlated with decreased silencing of reporter genes inserted near telomere proximal DNA elements. To reconcile the opposite observations in yeast and mammalian cells, several explanations can be considered. Firstly, mammalian aging is a lot more complex than yeast replicative aging and differences in the rate and mechanism of aging exist across tissues even within the same organism. In our studies using H4K16 acetylation analysis in ‘old’ wild type mice, H4K16 hypoacetylation could be observed in the liver, heart and bone marrow, but not in the kidney, for as yet unknown reasons. Mammalian Sirt1 (mammalian Sir2 homolog) function is itself pleiotropic and cell type-specific. Contradictory reports exist even within studies using mice as model systems because Sirt1 function was found pro-longevity in some studies, whereas in others Sirt1 was observed to induce senescence [69]. Also, our observation that sodium butyrate, a class I and II HDAC inhibitor could increase H4K16 acetylation, suggests that apart from Sirt1 (a class III deacetylase), other deacetylases also involved in the regulation of H4K16 acetylation levels in mammalian cells. Moreover, in contrast to yeast, mammalian Sirt1 has multiple substrates like Foxo3a and p53 [70], and some of these substrates may be of greater importance than histone H4 in aging. Lastly, similar to DNA methylation, where global DNA hypomethylation but site-specific hypermethylation is observed during aging [71], it is possible that the regulation of H4K16 acetylation might be more complex than what we currently understand.

## Epigenetic misregulation, DNA repair and aging: an emerging paradigm

A fundamental manner in which epigenetic misregulation in the form of DNA methylation and histone modification alterations can contribute to aging is by altering gene expression patterns. Since this has been the topic of discussion elsewhere [72], here we consider specific examples where epigenetic modifiers deregulated in aging may be involved in DNA repair. This point-of-view is especially important since the loss of DNA repair is a hallmark of aging and it is possible that epigenetic misregulation can contribute to aging by disrupting genome maintenance (summarized in Table 1).

**Table 1:** Histone modifications and chromatin remodelling proteins involved in double strand break repair and aging

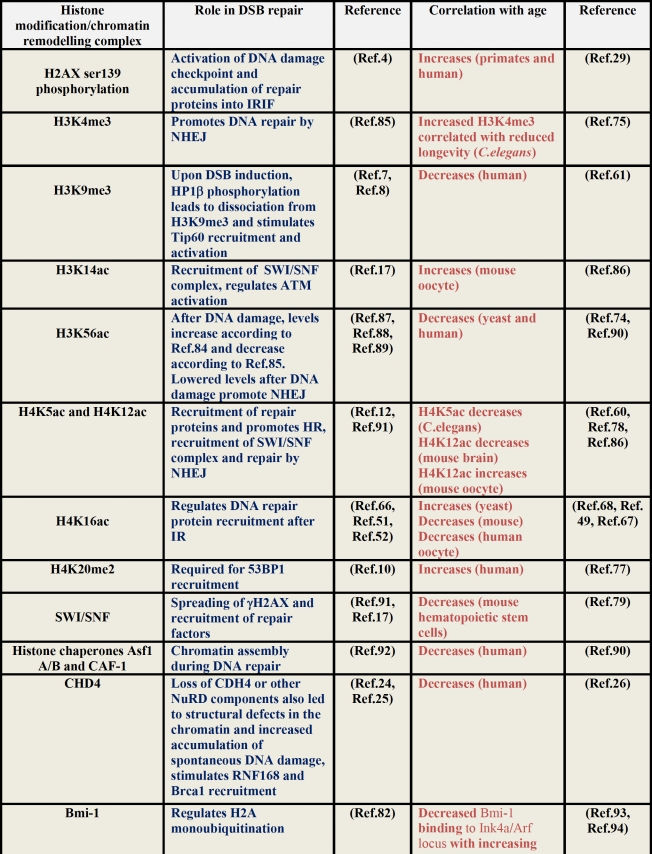

Through the use of model systems such as *S.cerevisiae*,*C.elegans*, mice and human cells, alterations in histone modifications and chromatin remodelling factors during aging have been studied. For example, *S.cerevisiae* cells undergo changes in histone levels and histone modifications with an increase in replicative age. As examples, total histone H3, H4 and H2A protein levels are greatly reduced in aged yeast cells relative to young cells [73]. Another example is the reduced expression of H3K56 acetylation with advancing age in *S.cerevisiae* cells [73, 74]. In *C. elegans*, members of the ASH-2 trithorax complex,ASH-2 itself, WDR-5 and the H3K4 methyltransferase SET-2 were found detrimental for longevity [75]. In another study, the acetylation of H4K5, but not total H4, was shown to decrease with age in *C.elegans*, and longevity could be extended with an HDAC inhibitor [60]. A global reduction in H4 acetylation has also been observed in rat cerebral cortex neurons and cultured human cells [76]. H4K20 trimethylation levels are found to increase with increasing age in human tissues and HGPS cells [77]. Recently the misregulation of H4K12 acetylation has been found to be associated with cognitive decline in mice models [78]. In a genome-wide expression analysis using highly purified hematopoietic stem cells from ‘young’ and ‘old’ mice, a functional deficit in stem cell function with advancing age was noted. Interestingly, an age-dependent reduction in the expression of subunits in the SWI/SNF, and HDAC 1, 5, and 6, were observed in this study [79]. In an interesting screen, the MRG/MORF family of proteins that are components of both HATS and HDAC complexes were identified as being involved in cell senescence and proposed to be regulators of aging [80]. Another commonly identified hallmark of aging cells is the loss of H3K9 trimethylation, which leads to loss of heterochromatin [39, 61]. In a recent study, two histone binding proteins RBBP4 and RBBP7, were found reduced in HGPS cells and were reported responsible for DNA damage accumulation. It was suggested that declining NURD remodelling complex functions in aging-associated chromatin defects and accumulation of DNA damage during aging [26]. The loss of SIRT6, a H3K9 deacetylase leads to premature aging-like phenotypes in mice, which was attributed to the ability of Sirt6 to regulate telomere function, aging-associated gene expression programs and to mobilize DNA-PK catalytic subunit (DNA-PKcs) to chromatin in response to DNA damage [81].Lastly, Bmi1 belongs to the polycomb complex of proteins that are required for H3K27 trimethylation. Although the role of Bmi1 is associated with aging primarily due to its ability to repress the *Ink4a/Arf* locus, it is now come to light that Bmi1 can also maintain genomic integrity by regulating H2A ubiquitination in response to DNA damage [82]. In summary, there is sufficient evidence that histone modifications/chromatin remodelling proteins involved in the maintenance of genomic integrity are misregulated during aging. What is not fully understood is to what extent this altered epigenetic landscape contributes to defective DNA repair during aging. This is because aging is complex and multifactorial, and thus any model assuming a linear relationship between epigenetic status, DNA repair and accelerated aging might be too simplistic and difficult to investigate experimentally. Indeed, just as defective chromatin modifications may impair DNA repair and cause the accumulation of DNA damage to trigger premature aging, the reverse has also been demonstrated. For example, aging-associated redistribution of chromatin modifiers such as Sirt1 to sites of DNA damage leads to changes in gene expression, which can once again alter the consequence of DNA repair [83]. Another example is the age-associated decline in histone chaperone levels which can alter chromatin structure to cause defective DNA repair [84]. Thus, the interplay between chromatin modification, DNA repair and aging is not straightforward and more studies with better model systems are required to understand the interrelation-ships.

## Conclusions and future directions

Although it is known that the loss of genomic integrity is an important hallmark of aging, the molecular mechanisms are only beginning to be understood. Through the use of Zmpste24-knock mice as a model to understand premature aging, interesting insights have been obtained on how genomic integrity can be regulated by epigenetic mechanisms such as histone acetylation. H4K16 hypoacetylation affects global chromatin structure to impair DNA damage recognition and repair and this, in turn contributes to genomic instability during aging. Recent findings have indicated that the study of Zmpste24-null mice may have broader relevance in understanding physiological aging. Therefore, it would be interesting to find out if histone acetylation levels can also affect genomic integrity during normal aging. Given the inherent reversibility of epigenetic modifications, it would then be possible to manipulate histone acetylation levels and relax chromosome structure to promote DNA repair during normal aging and restrain some age-associated pathologies.

In conclusion, a complete understanding on how exactly chromatin modifiers modulate DNA repair and aging at the molecular level is far from complete. Indeed, we are only beginning to see the ‘tip of the iceberg’, as far as chromatin, DNA repair and aging are concerned. It is anticipated that through the use of modern genome-wide technologies and mouse genetics, the study of epigenetics and aging will gain greater momentum.

## Nonstandard abbreviations

DSB: double strand break
AcH4K16: acetylated lysine 16 of histone H4
IRIF: irradiation-induced foci
HR: homologous recombination
NHEJ: non-homologous end joining
HAT: histone acetyltransferase
HDAC: histone deacetylase
MEF: mouse embryonic fibroblast
HGPS: Hutchinson Gilford progeria syndrome
IR: Irradiation
AT: Ataxia-telangiectasia
